# Juvenile Songbirds Compensate for Displacement to Oceanic Islands during Autumn Migration

**DOI:** 10.1371/journal.pone.0017903

**Published:** 2011-03-25

**Authors:** Kasper Thorup, Troels Eske Ortvad, Jørgen Rabøl, Richard A. Holland, Anders P. Tøttrup, Martin Wikelski

**Affiliations:** 1 Center for Macroecology, Evolution and Climate, Zoological Museum, University of Copenhagen, Copenhagen, Denmark; 2 Department of Migration and Immuno-ecology, Max Planck Institute for Ornithology, Radolfzell, Germany; 3 Department of Biology, Center for Macroecology, Evolution and Climate, University of Copenhagen, Copenhagen, Denmark; Institut Pluridisciplinaire Hubert Curien, France

## Abstract

To what degree juvenile migrant birds are able to correct for orientation errors or wind drift is still largely unknown. We studied the orientation of passerines on the Faroe Islands far off the normal migration routes of European migrants. The ability to compensate for displacement was tested in naturally occurring vagrants presumably displaced by wind and in birds experimentally displaced 1100 km from Denmark to the Faroes. The orientation was studied in orientation cages as well as in the free-flying birds after release by tracking departures using small radio transmitters. Both the naturally displaced and the experimentally displaced birds oriented in more easterly directions on the Faroes than was observed in Denmark prior to displacement. This pattern was even more pronounced in departure directions, perhaps because of wind influence. The clear directional compensation found even in experimentally displaced birds indicates that first-year birds can also possess the ability to correct for displacement in some circumstances, possibly involving either some primitive form of true navigation, or ‘sign posts’, but the cues used for this are highly speculative. We also found some indications of differences between species in the reaction to displacement. Such differences might be involved in the diversity of results reported in displacement studies so far.

## Introduction

There is strong evidence that migratory birds inherit an endogenous directional programme, steering inexperienced migrants in a certain direction for a certain period of time [1], [2]. This programme alone does not enable migrants to navigate toward their unknown species-specific wintering grounds and thus, it does not allow birds to compensate for a displacement [3]. With experience, this programme develops into a goal area navigation programme allowing the birds to accurately pin-point at least their breeding and winter grounds. Why this is so remains poorly understood [4]. Being able to compensate for displacements would presumably lead to evolutionary advantages but these have apparently not led to the evolution of such a capability in young birds, possibly because of evolutionary or mechanistic constraints but also because juveniles may benefit from a more open strategy.

The presumed change from a simple bearing-based orientation to true navigation is largely unstudied. It is generally assumed that the development of the navigational system in adult birds, which enables accurate goal navigation, relies on experience during the first migration [5], [6]. In general, juvenile birds making their first migration do not show signs of adult navigation after release in experimental displacements where the compensation requires some form of true navigation [7], [8]. Despite this, there are no theoretical objections to juvenile migrants being able to correct for displacements: Juveniles could correct by using experienced-based navigating toward a site already visited on migration, as birds already on the way on their first migration could be acquiring information necessary for later navigation.

The way the initial programme interacts with and is influenced by external factors such as wind, topography, habitat etc. is still largely unknown. For example, winds can exert a strong influence on migrating birds, because wind speeds can easily reach the endogenous self-propelled flight speed of most birds. A few studies have indicated an ability at the individual level to compensate for wind displacements [9], [10], whereas others have not [11]. In juvenile birds with no prior knowledge of the migratory route or wintering grounds, the behavioural responses to such displacements are crucial for the successful arrival at the species-specific wintering grounds.

Here, we study naturally (ie. likely by wind) and experimentally displaced juvenile birds of nocturnally and solitarily migrating species at the Faroe Islands in the northeastern Atlantic Ocean far off the normal migration route of European land birds. So far responses to displacements and especially to external factors have proven very difficult to study, mainly because of problems in following free-flying migratory birds [12]. Thus, we applied advanced radio telemetry methods to efficiently track the movements of departing birds after the displacement to the Faroe Islands and combined this with the more traditional orientation cage studies.

## Results

**Figure 1:**
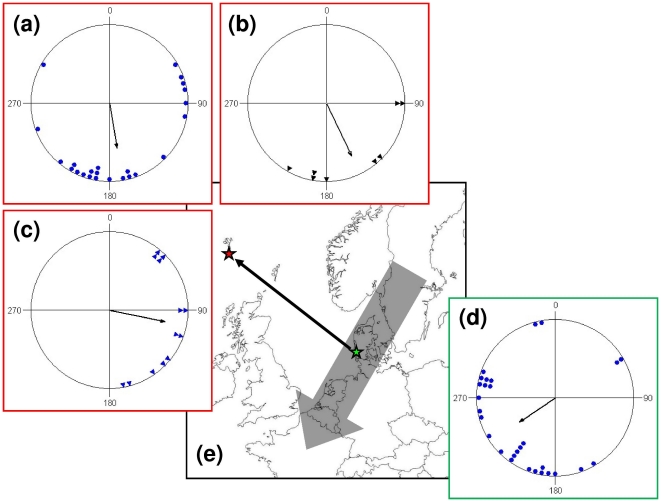
Orientation of juvenile birds displaced to the Faroe Islands. (a–c) Orientation on the Faroe Islands: (a) in funnels of birds displaced from Denmark to the Faroe Islands, (b) vanishing bearings of birds caught on the Faroe Islands, (c) vanishing bearings of birds translocated from Denmark to the Faroe Islands. (d) Orientation in funnels of birds caught and tested in Denmark. (e) Map of Northwest Europe showing the location of the Faroe Islands (red star) and the main migration through Northwest Europe (grey arrow). The 1100 km displacement from Denmark (green star) to The Faroes is shown by the thin arrow. In circular diagrams, the orientation of individual birds is marked on the periphery of the circle and the mean sample orientation is shown as a black arrow starting in the circle centre and with its length relative to the radius corresponding to the length of the mean vector *r*. 0 corresponds to orientation toward North. All sample orientations differ significant from random according to the Rayleigh test (*P*<0.05).

**Figure 2:**
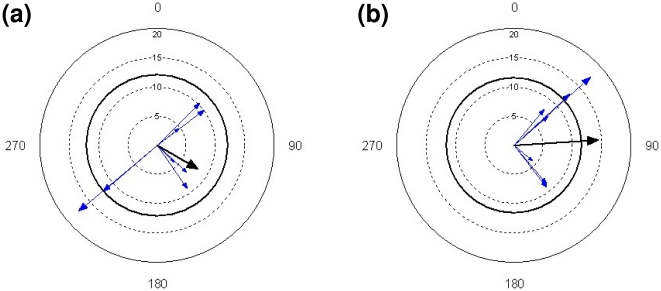
Strength (m/s) and direction (°) of the wind vector at each departure from the Faroe Islands of birds caught on the Faroes (a) and in Denmark (b). North is zero degress. The thick arrow shows the direction of the mean wind vector and the thick circle the 5% level for significance of the mean vector according to the Rayleigh test. Note that winds are given as vectors, i.e. a vector pointing southeast corresponds to what would normally be termed northwesterly winds. On average, birds took off in tail winds but the mean of winds during take-offs was not different from random in naturally displaced birds whereas experimentally displaced birds had on average winds blowing toward East (*P*<0.01) when migrating.

Juvenile birds of three long-distance migratory species, experimentally displaced 1100 km from Denmark to the Faroe Islands, shifted their orientation similarly and in accordance with compensation for the displacement. Before displacement, juvenile birds tested in Emlen funnels in Denmark were oriented toward southwest (α = 236°, *r* = 0.544, *N* = 29, *P*<0.001, Rayleigh test; Figure 1b) similar to the normal migration direction as found from ring recoveries (*P*>0.7, Watson-Williams test). After displacement, the orientation in funnels on the Faroes shifted counter-clockwise toward south-southeast (α = 168°, *r* = 0.516, *N* = 25, *P*<0.001; Figure 1c) significantly different from the orientation in Denmark before displacement (*N* = 29/25, *F*_1,52_ = 12.88, *P*<0.001, WW test). Radio tracking of the displaced birds later released on the Faroes revealed departure directions that were significantly different from the orientation before displacement (*N* = 29/13, *F*_1,40_ = 40.18, *P*<0.001, WW test) and in even more counter-clockwise shifted directions toward southeast and east (α = 102°, *r* = 0.711, *N* = 13, *P*<0.001; Figure 1d) than in the orientation cages (*N* = 25/13, *F*_1,36_ = 10.01, *P* = 0.003, WW test; though the difference was not significant at level of the individual: α = 2°, *r* = 0.415, *N* = 11, *P*>0.05, Confidence Interval test). This difference between funnel orientation and departure directions could have been caused by winds, with birds on average departing in westerly tailwinds and with the average tailwind vector significantly different from random only when experimentally displaced birds departed (Figure 2). Furthermore, the overall pattern of headings was similar to that of vanishing bearings and headings were not more constant. Testing changes in orientation at the individual level also resulted in very similar results with the orientation after displacement differing significantly from the orientation in Denmark both for funnel tests and vanishing bearings (*P*<0.05 and 0.01, respectively, Confidence Interval test; Figure S1). Because of the relatively large scatter, it was not possible to determine whether orientation back toward the capture site or toward the winter grounds fitted the data best (Figure S2).

Similarly, birds naturally displaced to the Faroe Islands, presumably by wind drift, and followed with radio telemetry, apparently compensated for the natural displacement by departing in directions toward south-southeast (α = 155°, *r* = 0.734, *N* = 8, *P* = 0.009, Rayleigh test; Figure 1e). The direction differed significantly from that found in birds caught and tested in cages within the normal migration route in Denmark (*N* = 8/29, *F*_1,35_ = 8.46, *P* = 0.006, WW test) and also from the departure directions of experimentally displaced birds (*N* = 8/13, *F*_1,19_ = 5.75, *P* = 0.027, WW test) but not from the orientation of displaced birds in funnels.

In general, no differences among species were apparent. Only if combining naturally and experimentally displaced birds, the vanishing bearings from the Faroe Islands differed slightly among species (*N* = 11/3/7, *F*_2,18_ = 4.11, *P* = 0.034, WW test). Garden warblers *Sylvia borin* took off in a more southerly direction (α = 181°, *r* = 0.939, *N* = 3, *P* = 0.058) than both willow warblers (α = 93°, *r* = 0.797, *N* = 7, *P* = 0.007) and blackcaps (α = 124°, *r* = 0.663, *N* = 11, *P* = 0.005). The differences in funnel orientation and headings among species were smaller and not significant.

## Discussion

The compensatory reaction in drifted juvenile birds could be caused by reverse path integration (dead-reckoning) to register the passive displacement or an even simpler non-specific reaction to westward displacement by winds during a migratory flight as in corrective early morning flights [13], [14]. Directional compensation does not need to be a very precise mechanism but could be a non-specific reaction to any westward displacement. Despite the expectations from previous releases of displaced birds, the additional compensation by experimentally displaced birds indicates surprisingly, that in some circumstances, juveniles may be able to correct for displacements from the normal route. The juvenile passerines observed here might well have used a simple form of true navigation.

The navigation of birds on the Faroes could have been based on an experience-based system that was initiated earlier than is generally assumed in these or other species. The tracks taken by juvenile Eleonora's falcons suggest the possibility of goal-area navigation toward the winter grounds independent of the adults [15] and such restricted migration has also been shown in several other species [16].

Previously, only a few studies of bird migrants have hinted at the existence of some form of navigational responses in juveniles [8], [17], in contrast to a number of studies showing no compensation [2], [19], [20]. However, in juvenile turtles changes in orientation as a response to experimental changes of the magnetic field may indicate the use of an inborn navigational system based on magnetic ‘sign post’ cues [21]–[23] similarly to the apparently inborn responses to magnetic field changes reported in birds where fat deposition needed for crossing an ecological barrier is apparently partly controlled by magnetic cues [24], [25].

Our results indicate that there could be minor differences in reactions among species. The reasons for such differences might relate to different motivational states and differences in migration routes and distances, but warrant further in-depth studies. Blackcaps tested on the Faroes by Rabøl [26] were oriented in the normal migration direction of the Norwegian population in contrast to the pattern seen here and such a difference could easily be the result of even small differences in conditions on arrival between years. The expected evolutionary advantages associated with being able to compensate for displacements are not fully obvious because juveniles may benefit from dispersing longer than adults as well as from a bet-hedging strategy with regard to migratory directions [27]. In this study, the perhaps most pronounced long-distance migrant, the garden warbler, showed the least reaction to the displacement. Though no firm conclusions can be drawn due to small sample sizes of this species, this could be because for garden warbler the direction to a far winter quarter changes less than in the shorter-distance migrants or be because immediate compensation for displacement is not necessarily optimal when making optimal use of winds to reach a goal far away [28].

Magnetic cues seems to be the most obvious candidates for forming the basis of the navigational responses to the displacement [29], [30] with relatively large magnetic field changes for the distances involved in this study (change in declination: 9°; dip: 4°; magnetic intensity: 1500 nT). Inexperienced migrants have been shown to increase fattening and change their innate directional preference in response to changes in the magnetic field [25], [31] but the involvement of the magnetic field in migratory navigation remains unresolved. Experiments with silvereyes and northern wheatears have shown responses indicating that the magnetic field can be used for navigation [32], [33]. Other cues such as celestial [18] or olfactory cues [34] may also be involved.

Given the restricted range of the tracking methods used here, the ultimate fate of the displaced birds is not known. To be able to find out whether birds are able to find their normal winter grounds would require being able to track the full migrations of these birds. We believe that the possibility of further experimentation with free-flying birds will result in much improved possibilities for investigating the complex relationship with the environment and that such experimentation is likely to enable us to understand the fascinating navigational mechanisms in long-distance migrating birds. With the development of smaller tracking devices this appears possible in a near future.

## Materials and Methods

### Ethics statement

This study was carried out in strict accordance with Guidelines to the use of wild birds in research of the Ornithological Council [35]. Animal work was approved by the Danish Forest and Nature Agency by permission to the Copenhagen Bird Ringing Centre (J.nr. SN 302-009) and the Faroese Food and Environmental Agency (J.nr. 2009-00101-43).

### Study species

We studied the orientation of juvenile individuals of three small, passerines – blackcap *Sylvia atricapilla*, garden warbler and willow warbler *Phylloscopus trochilus* weighing from around 10 g in the willow warbler to around 20 g in blackcap and garden warbler. In general, the birds migrate southwest from northwest Europe with Denmark located in the main migration corridor and the Faroe Islands far off (Figure 1a). For the three species, the average direction from ringing to recovery sites is very similar. In blackcaps it is 198°, in garden warbler 204° and in willow warbler 196°. The species are nocturnal migrants breeding commonly in northern Europe and generally spending their non-breeding seasons in sub-Saharan Africa though some blackcaps winter in the Mediterranean region. Because overall migration route and wintering grounds are similar, it was considered acceptable to pool the species for analyses.

### Orientation

We investigated birds experimentally displaced 1 100 km from Denmark to the Faroe Islands as well as birds naturally displaced to the Faroe Islands during autumn 2009.

For the experimental displacement, 31 birds were caught 1–10 September during westerly winds at Blåvand, Denmark (55.33°N; 8.06°E). Unfortunately, it was not possible to test the birds locally because of hunting. Instead, the birds were kept in cages with food and water ad libitum before being transported 140 km to Endelave, Denmark (55.45°N; 10.19°E) where their orientation was tested under the starry sky on 11 and 13 September. The birds were then displaced to the Faroe Islands by plane from Copenhagen Airport on 16 September. Their orientation was tested in funnels on starry nights 22, 23 and 27 September on the southernmost tip of the Faroes (Akraberg). After orientation tests, the birds were fitted with radio transmitters and released in Sumba.

For the natural experiment, 21 birds were caught in mist nets 11 September to 11 October in Sumba village (61.24°N; 6.42°W) with two additional individuals caught in Nólsoy (62.00°N; 6.40°W) and transported in cages to Sumba. After capture (or after transport from Nólsoy), birds were fitted with radio transmitters and released during daylight.

Because the number of orientation tests that could be carried out simultaneously on the Faroes was limited, only individuals experimentally displaced from Denmark were tested in Emlen funnels. At the Danish test site, no lights are visible on the sky from the test cages. Apart from a lighthouse to the east-southeast which was not in view from the test site due to a lower elevation (and not lighting in the direction of the test site), there are no visible artificial light sources at all at the Faroese test site. The orientation was tested both before and after displacement. Funnel tests were carried out under clear skies with no moon, approximately two hours after sunset and lasting approximately 1½ hour. Funnels were painted inside with whitewash and the orientation was estimated from the scratches left [36]. We only included orientation if there were at least 30 scratches and the activity pattern was mono-modal or, in the few cases where a bird showed bimodal orientation, one peak was clearly larger than the other. Only one test was included for each bird. In general, birds were tested only once, but five birds were tested on two different days on the Faroes. If birds were clearly oriented on the first day only this orientation was included in analyses. In total, 29 birds were well oriented in funnels in Denmark and 25 birds on the Faroes (see Table S1). For one bird, no directions were obtained from Denmark or the Faroes.

For all birds, both experimentally and naturally displaced, we tested the migratory orientation when the birds departed from the Faroe Islands 11 September to 14 October. For this purpose, we glued 0.4-g radio transmitters (LB-2N, Holohil Systems Ltd) to the backs using eyelash adhesive. The signals from the radio transmitters were recorded manually with a handheld antenna and automatically tracked by two automatic receiver stations with three antennas pointing West, South and East, respectively. This arrangement of the automatic receiver stations allowed us to follow departures in all directions with the poorest coverage of departures in northerly directions which are expected to be rare during autumn. One receiver station was placed above the village (Sumba) overlooking the release area and one located at the highest point to the south at Akraberg. The orientation of the migrants was estimated from the bearings at the last point before the radio signal dropped to noise level (vanishing bearings; [37]). Bearings were obtained manually and crosschecked with the data from automatic receiver stations. It seems unlikely that the directions observed for the birds in this study should be the result of site-specific bias due to the fact that another group of birds followed by radio telemetry during the study period but consisting of other species with southeast normal migration directions departed in overall westerly directions very different from the directions reported here (Thorup et al. *in prep*).

**Figure 3:**
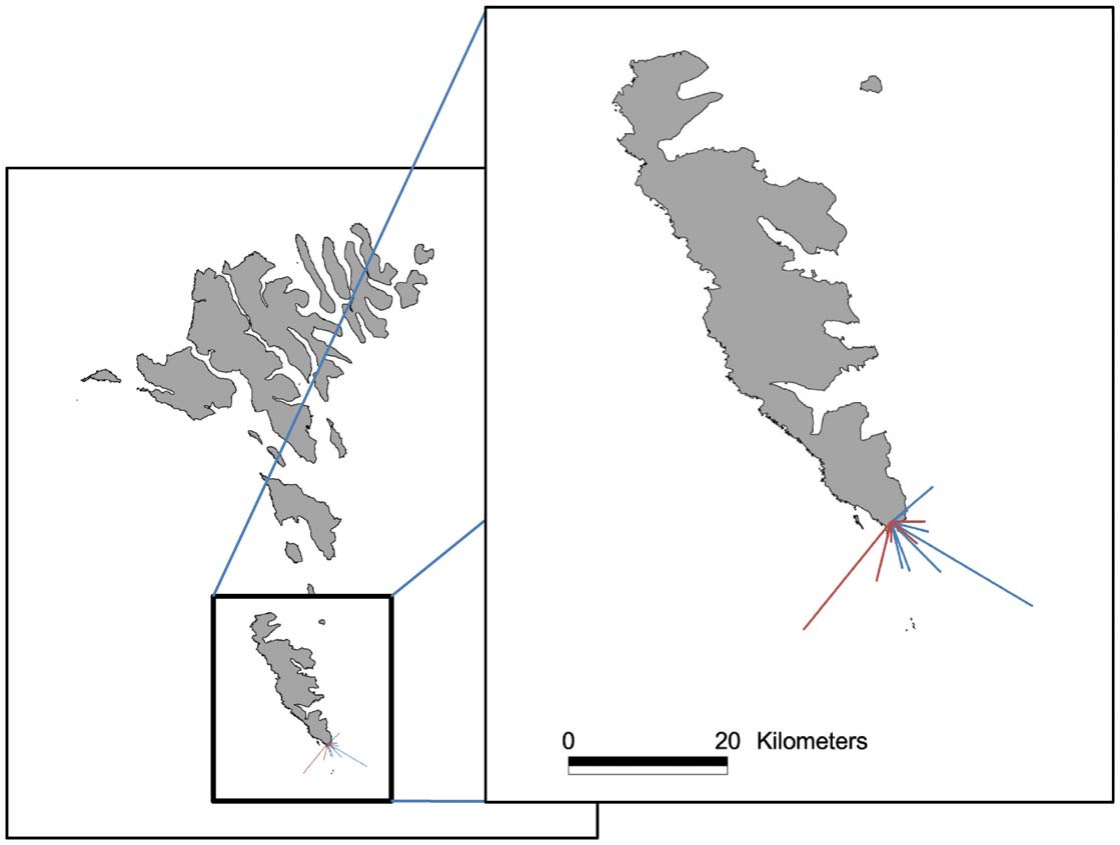
Reconstructed tracks of radio tagged migrants departing from Sumba, the southernmost point of the Faroe Islands. Red lines  =  presumably wind displaced migrants (N = 12), Blue lines  = translocated migrants (N = 8).

Migratory flights were recorded for 21 individuals (13 of the 31 birds experimentally displaced from Denmark and 8 of 23 birds caught on the Faroes). The migrants departed the village between 01:43 and 05:14 hrs after sunset (mean =  1:52). On average, birds were tracked for 22 minutes of which 12 minutes were estimated to be spent on migration after an initial, height-gaining take-off phase. Wind data from the weather station at Akraberg (Danish Meteorological Institute) were used to calculate headings following Åkesson [38]. Some birds took off in quite strong winds up to 17.5 m/s exceeding the birds' airspeed, but 16 birds took off in wind speeds less than 12 m/s with an overall average of 9.2 m/s. On average, birds took off in tail winds but the mean of winds during take-offs was not different from random in naturally displaced birds whereas experimentally displaced birds had on average south-easterly winds (*P*<0.01) when migrating (Figure S1). The intrinsic flight speed for all birds was set conservatively to 10 m/s resulting in one case where it was not possible to calculate a heading. Assuming a flight speed of 10 m/s for the portion of tracking when the birds appeared to be migrating after initial take off and correcting for winds, birds could be tracked up to 23 km with an average 7 km, meaning that most birds flew out over open ocean (Figure 3)

Using oriana vers. 3, we tested if the orientation of samples could be considered random using the Rayleigh test and differences in orientation between and among samples were tested with Watson-Williams test [39]. Differences between samples were also tested at the individual level using a confidence interval test on the individual differences.

## Supporting Information

Figure S1 **Differences between orientation in the Faroe Islands and Denmark.** Differences between the Emlen funnel orientation in Denmark and (a) the Emlen funnel orientation in the Faroes or (b) vanishing bearing on the Faroe Islands. The circular diagrams show differences at the individual level marked on the inside of the periphery. Mean differences are marked with a thick line from center of the circle and the corresponding 95% confidence intervals are also indicated.
(TIF)

Figure S2 **The displacement of juvenile birds to the Faroe Islands.** Map of Europe and West Africa showing the location of the Faroe Islands (red star) and the main migration through Northwest Europe to West Africa (grey arrow). The migration route and breeding and wintering grounds are similar for the three species studied. The 1100 km displacement from Denmark (green star) to the Faroes is shown by the thin arrow. Possible migration routes from the Faroes are shown as normal migration direction (1), toward wintering area (2), and back toward capture site (3).
(TIF)

Table S1 **Details of the experiments.**
(DOC)
