# Supplementary figures and images for: Juvenile Songbirds Compensate for Displacement to Oceanic Islands during Autumn Migration
Source: PLoS One. 2011 Mar 25;6(3):e17903. doi: 10.1371/journal.pone.0017903 (PMC3064565; doi:10.1371/journal.pone.0017903)

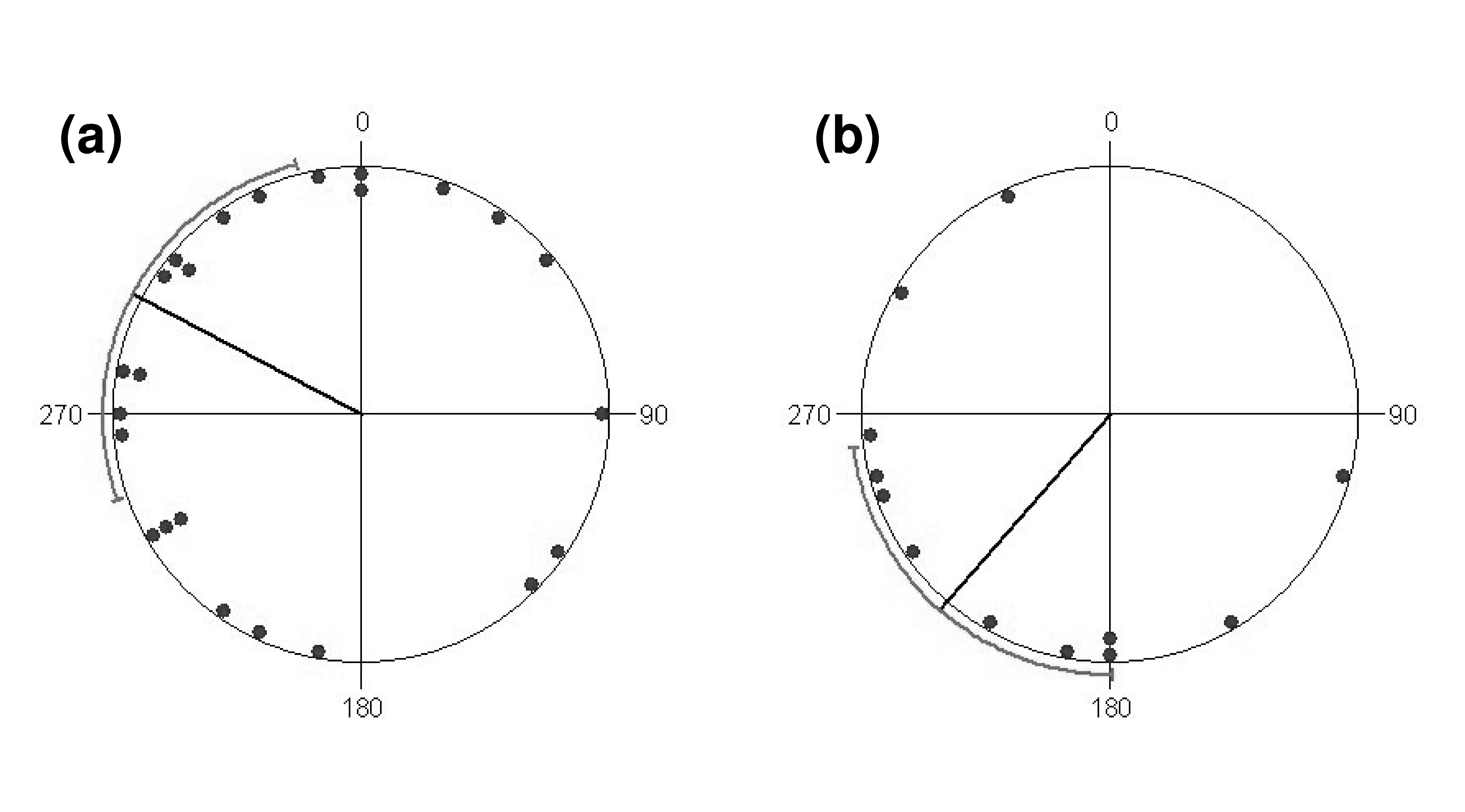

Supplement: Figure S1 — Differences between orientation in the Faroe Islands and Denmark. Differences between the Emlen funnel orientation in Denmark and (a) the Emlen funnel orientation in the Faroes or (b) vanishing bearing on the Faroe Islands. The circular diagrams show differences at the individual level marked on the inside of the periphery. Mean differences are marked with a thick line from center of the circle and the corresponding 95% confidence intervals are also indicated. (TIF) [file pone.0017903.s001.tif]

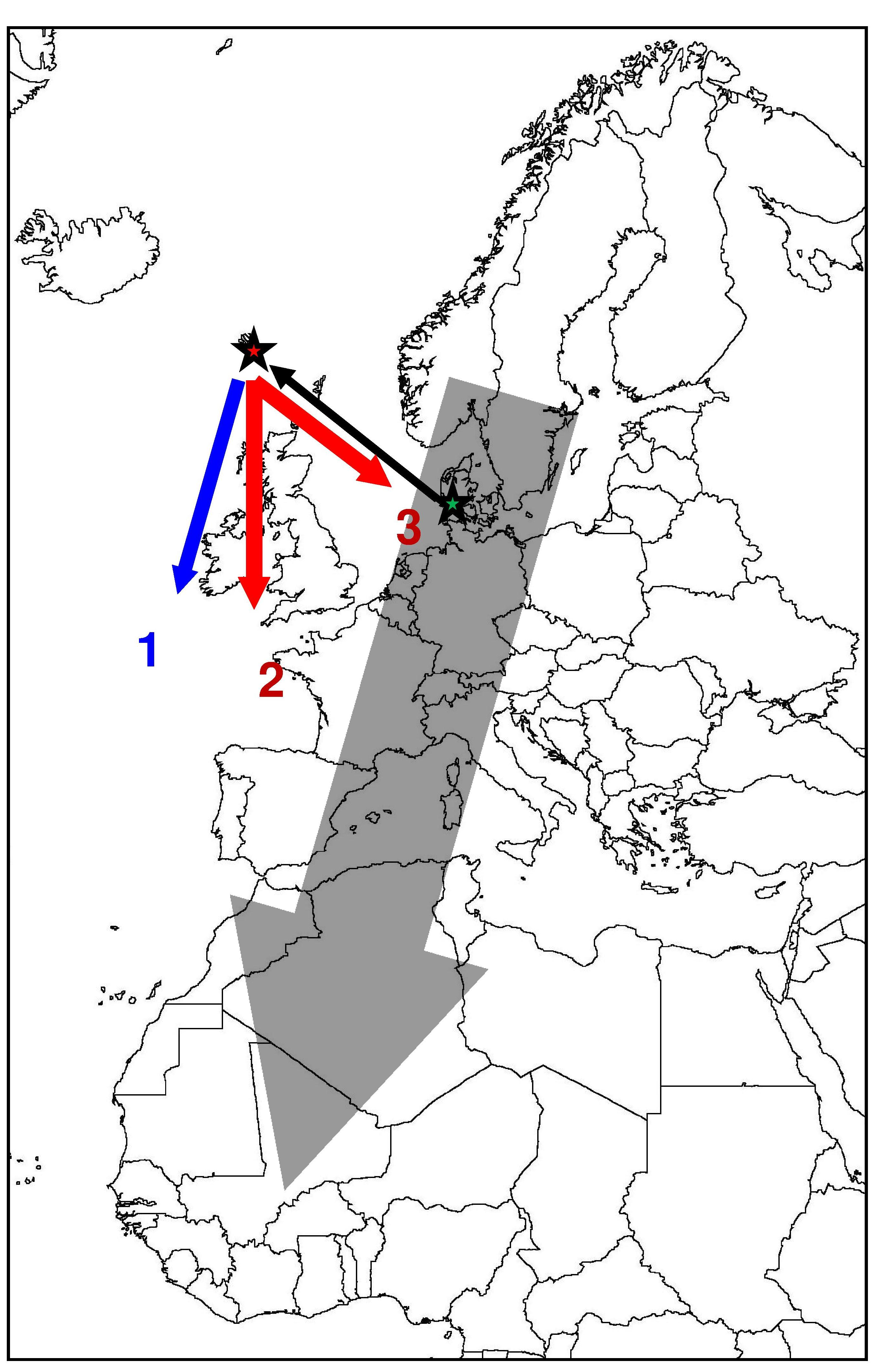

Supplement: Figure S2 — The displacement of juvenile birds to the Faroe Islands. Map of Europe and West Africa showing the location of the Faroe Islands (red star) and the main migration through Northwest Europe to West Africa (grey arrow). The migration route and breeding and wintering grounds are similar for the three species studied. The 1100 km displacement from Denmark (green star) to the Faroes is shown by the thin arrow. Possible migration routes from the Faroes are shown as normal migration direction (1), toward wintering area (2), and back toward capture site (3). (TIF) [file pone.0017903.s002.tif]
