# Supplementary material for: Juvenile Songbirds Compensate for Displacement to Oceanic Islands during Autumn Migration
Source: PLoS One. 2011 Mar 25;6(3):e17903. doi: 10.1371/journal.pone.0017903 (PMC3064565; doi:10.1371/journal.pone.0017903)
Supplement: Table S1 — Details of the experiments. (DOC) [file pone.0017903.s003.doc]

**Table S1. Details of the experiments.**

| ID | Species | Capture location | Orient, Funnel, Denmark (°) | Orient, Funnel, Faroes (°) | Vanish bear, Faroes (°) | Head, Faroes (°) | Release date, Faroes | Takeoff date | Wind dir (°) | Mean wind speed (m/s) | Track length (km) | Air time (min) | Migr time (min) |
| --- | --- | --- | --- | --- | --- | --- | --- | --- | --- | --- | --- | --- | --- |
| 565 | Phylloscopus trochilus | Denmark | 240 | 190 |  |  |  |  |  |  |  |  |  |
| P24562 | Phylloscopus trochilus | Denmark | 150 | 70 | 40 | 26 | 25/09 | 25/09 | 48 | 17.5 | 8.1 | 5 | 5 |
| P24566 | Phylloscopus trochilus | Denmark | 290 | 210 | 110 |  | 24/09 | 24/09 | 47 | 13.0 | NA | 20 | 5 |
| P24569 | Phylloscopus trochilus | Denmark | 190 |  | 40 | 26 | 25/09 | 25/09 | 48 | 17.5 | 8.1 | 15 | 5 |
| 650 | Sylvia atricapilla | Denmark | 285 | % |  |  |  |  |  |  |  |  |  |
| 666 | Sylvia atricapilla | Denmark | 280 | % |  |  |  |  |  |  |  |  |  |
| 667 | Sylvia atricapilla | Denmark | 280 | 135 |  |  |  |  |  |  |  |  |  |
| 670 | Sylvia atricapilla | Denmark | 215 | 165 |  |  |  |  |  |  |  |  |  |
| 673 | Sylvia atricapilla | Denmark | 260 | 25 |  |  |  |  |  |  |  |  |  |
| 681 | Sylvia atricapilla | Denmark | 285 | 195 |  |  |  |  |  |  |  |  |  |
| AX26638 | Sylvia atricapilla | Denmark | 215 | 60 | 110 | 85 | 30/09 | 30/09 | 140 | 8.6 | 4.9 | 20 | 5 |
| AX26644 | Sylvia atricapilla | Denmark |  | 80 | 130 | 121 | 30/09 | 30/09 | 140 | 8.6 | 23.1 | 21 | 21 |
| AX26654 | Sylvia atricapilla | Denmark | 195 | 170 | 135 | 185 | 23/09 | 23/09 | 49 | 7.7 | 1.2 | 3 | 3 |
| AX26655 | Sylvia atricapilla | Denmark | 185 | 90 | 90 | 45 | 01/10 | 03/10 | 142 | 9.0 | 3.0 | 4 | 4 |
| AX26658 | Sylvia atricapilla | Denmark | 270 | 100 | 145 | 195 | 23/09 | 23/09 | 49 | 7.7 | 10.8 | 63 | 32 |
| AX26674 | Sylvia atricapilla | Denmark | 345 | 75 | 90 | 75 | 28/09 | 28/09 | 130 | 4.1 | 3.8 | 5 | 5 |
| AX26684 | Sylvia atricapilla | Denmark | 255 | 200 | 45 | 21 | 28/09 | 28/09 | 130 | 4.1 | 2.8 | 14 | 5 |
| AX26687 | Sylvia atricapilla | Denmark | 225 | 190 |  |  | 01/10 | 01/10 |  |  |  |  |  |
| AX26689 | Sylvia atricapilla | Denmark | 215 | 250 | 45 | 21 | 28/09 | 28/09 | 130 | 4.1 | 2.8 | 40 | 5 |
| 641 | Sylvia borin | Denmark | 285 | 165 |  |  |  |  |  |  |  |  |  |
| 651 | Sylvia borin | Denmark | 180 | 180 |  |  |  |  |  |  |  |  |  |
| 693 | Sylvia borin | Denmark | 215 | 205 |  |  |  |  |  |  |  |  |  |
| 698 | Sylvia borin | Denmark | 215 |  |  |  |  |  |  |  |  |  |  |
| ID | Species | Capture location | Orient, Funnel, Denmark (°) | Orient, Funnel, Faroes (°) | Vanish bear, Faroes (°) | Head, Faroes (°) | Release date | Takeoff date | Wind dir (°) | Mean wind speed (m/s) | Track length (km) | Air time (min) | Migr time (min) |
| AX26648 | Sylvia borin | Denmark | 160 | 210 |  |  | 28/09 | 28/09 |  |  |  |  |  |
| AX26652 | Sylvia borin | Denmark | 280 | 160 |  |  | 25/09 | 25/09 |  |  |  |  |  |
| AX26661 | Sylvia borin | Denmark | 200 | 220 |  |  | 25/09 |  |  |  |  |  |  |
| AX26669 | Sylvia borin | Denmark | 350 |  | 170 | 208 | 23/09 | 23/09 | 40 | 8.0 | 8.3 | 56 | 50 |
| AX26676 | Sylvia borin | Denmark | 190 | 190 | 165 | 206 | 23/09 | 23/09 | 40 | 8.0 | 8.9 | 54 | 50 |
| AX26679 | Sylvia borin | Denmark | 60 | 195 |  |  | 24/09 | 24/09 |  |  |  |  |  |
| AX26680 | Sylvia borin | Denmark | 60 | 300 |  |  | 28/09 | 28/09 |  |  |  |  |  |
| P45003 | Phylloscopus trochilus | Faroes | % | % | 90 | 107 | 11/09 | 13/09 | 51 | 4.7 | 4.0 | 20 | 5 |
| P45005 | Phylloscopus trochilus | Faroes | % | % | 90 | 136 | 13/09 | 15/09 | 45 | 10.2 | 4.2 | 37 | 5 |
| P45006 | Phylloscopus trochilus | Faroes | % | % | 135 | 217 | 14/09 | 22/09 | 53 | 10.0 | 1.7 | 10 | 10 |
| P45008 | Phylloscopus trochilus | Faroes | % | % | 140 | 145 | 24/09 | 01/10 | 133 | 6.8 | 5.0 | 13 | 5 |
| AX41607 | Sylvia atricapilla | Faroes | % | % | 190 | 210 | 01/10 | 01/10 | 135 | 4.1 | 10.6 | 15 | 15 |
| AX41608 | Sylvia atricapilla | Faroes | % | % | 190 | 230 | 04/10 | 06/10 | 145 | 9.0 | 4.2 | 10 | 5 |
| AX41612 | Sylvia atricapilla | Faroes | % | % | 180 | 113 | 07/10 | 07/10 | 230 | 12.0 | 3.5 | 16 | 5 |
| AX41610 | Sylvia borin | Faroes | % | % | 210 | 173 | 07/10 | 07/10 | 230 | 17.5 | 22.0 | 20 | 15 |

Abbreviations: Blank fields indicate that no data were obtained, i.e. a bird did not take off, or was inactive or disoriented in an orientation cage. "%" in Orientation indicates that a bird was not tested (Note that birds captured on the Faroe Islands were not tested in funnels). Phylloscopus trochilus = willow warbler; Sylvia atricapilla = blackcap; Sylvia borin = garden warbler.
